# Supplementary material for: Sex Biases in Cancer and Autoimmune Disease Incidence Are Strongly Positively Correlated with Mitochondrial Gene Expression across Human Tissues
Source: Cancers (Basel). 2022 Nov 29;14(23):5885. doi: 10.3390/cancers14235885 (PMC9736300; doi:10.3390/cancers14235885)
Supplement: Supplementary file 1 [file cancers-14-05885-s001.zip › cancers-1948266 Supplementary Figures S1-S5.pdf]

Supplementary Figures for "Sex biases in cancer and autoimmune disease incidence are strongly positively correlated with mitochondrial gene expression across human tissues"

David R. Crawford[a,b], Sanju Sinha[a], Nishanth Ulhas Nair[a], Bríd M. Ryan[c], Jill S. Barnholtz-Sloan[d,e], Stephen M. Mount[b], Ayelet Erez[f], Ken Aldape[g], Philip E. Castle[e,h], Padma S. Rajagopal[a,i], Chi-Ping Day[j], Alejandro A. Schäffer[a] & Eytan Ruppin[a,*]

[a] Cancer Data Science Laboratory, Center for Cancer Research, National Cancer Institute, National Institutes of Health, Bethesda, MD

[b] Department of Cell Biology and Molecular Genetics, University of Maryland, College Park, MD

[c] Laboratory of Human Carcinogenesis, Center for Cancer Research, National Cancer Institute, National Institutes of Health, Bethesda, MD

[d] Center for Biomedical Informatics and Information Technology, National Cancer Institute, National Institutes of Health, Bethesda, MD

[e] Trans-Divisional Research Program, Division of Cancer Epidemiology and Genetics, National Cancer Institute, National Institutes of Health, Bethesda, MD

[f] Department of Biological Regulation, Weizmann Institute of Science, Rehovot, Israel

[g] Laboratory of Pathology, Center for Cancer Research, National Cancer Institute, National Institutes of Health, Bethesda, MD

[h] Division of Cancer Prevention, National Cancer Institute, National Institutes of Health, Bethesda, MD

[i] Women's Malignancies Branch, Center for Cancer Research, National Cancer Institute, National Institutes of Health, Bethesda, MD

[j] Laboratory of Cancer Biology and Genetics, Center for Cancer Research, National Cancer Institute, National Institutes of Health, Bethesda, MD

[*]Corresponding author, eytan.ruppin@nih.gov

Description of Supplementary Figures

Figure S1. The correlation between incidence rate sex bias for cancers and AIDs (using low-resolution cancer classification).

Figure S2. The correlation between incidence rate sex biases for cancer types and AIDs (using high-resolution cancer classification).

Figure S3. The correlation between checkpoint gene expression sex bias versus AID and cancer incidence rate sex bias.

Figure S4. The correlation between XIST gene expression versus AID and cancer incidence rate sex bias.

Figure S5. The correlation between mitochondrial gene expression versus AID and cancer incidence rates in males and females.

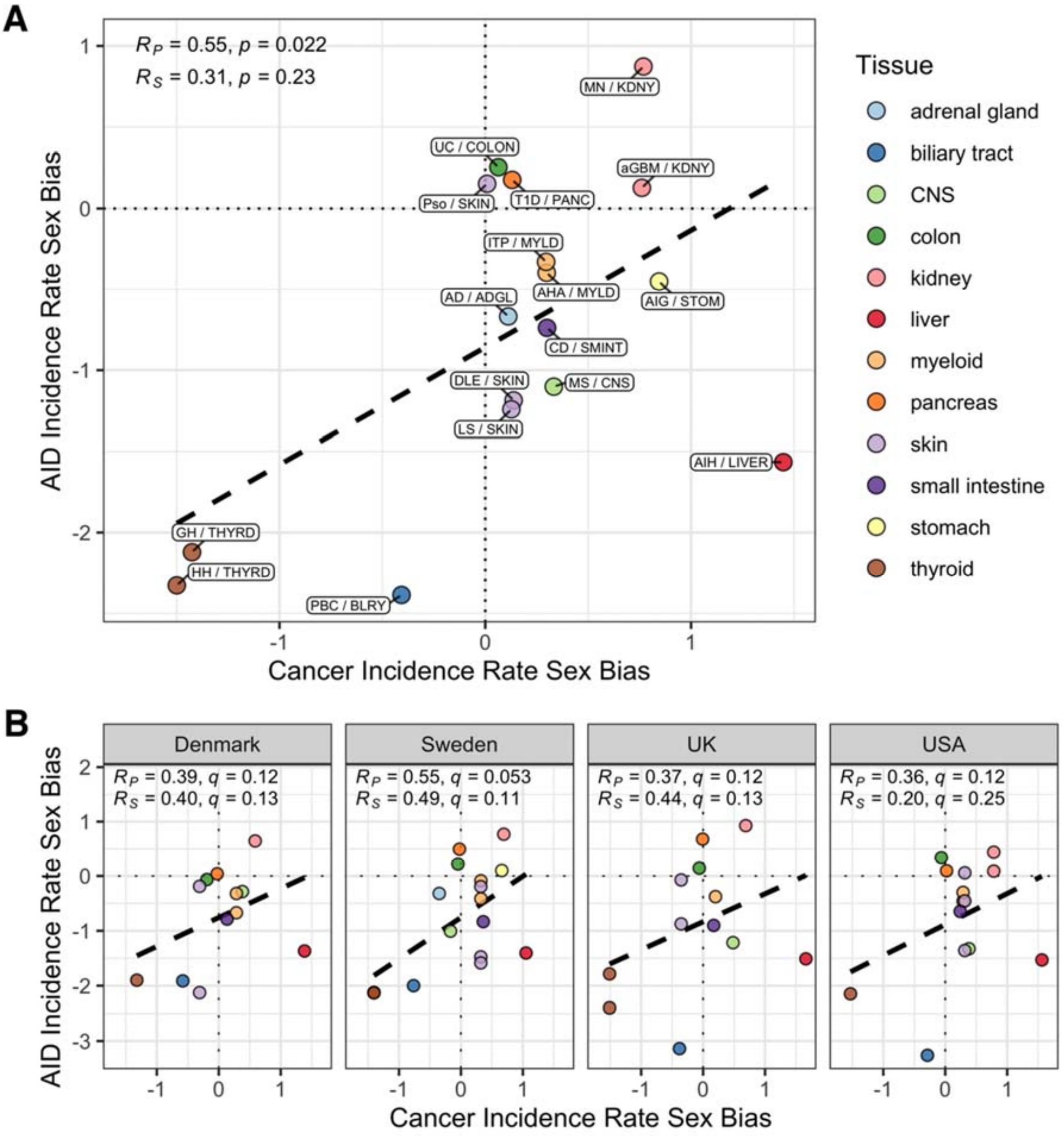

Figure S1. The correlation between incidence rate sex biases for cancers and AIDs (using low-resolution cancer type classification). Low-resolution cancer grouping divides cancers into 12 types. Tissue-matched incidence rate sex biases for cancers (X-axis) and for autoimmune diseases (Y-axis) are displayed across different tissues of origin (circle color indicates the tissue). Positive values in each of the axes indicate male bias; negative values indicate female bias. The dashed line is the linear regression line. Statistics in the top left corner include Pearson's product-moment correlation r-value (R_p) and a t-test p-value (2-sided for A, 1-sided for B); and the Spearman's rank correlation coefficient value (R_s) and a t-test p-value (2-sided for A, 1-sided for

B). For country-level tests, p-values were corrected for multiple testing using the Benjamini-Hochberg method to produce q-values. (**A**) Across-population averages, with the cancer-AID pairs labeled. (**B**) Population-level data for the 4 countries with the largest numbers of data pairs (at least 13 out of 17 cancer-AID pairs), maintaining the tissue color labels used in the top panel (Sweden, 17 pairs; USA, 15 pairs; Denmark & UK, each 13 pairs). **AIDs**: AD, Addison's disease; aGBM, anti-glomerular basement membrane nephritis; AHA, Autoimmune hemolytic anemia; AIG, Autoimmune gastritis; AIH, Autoimmune hepatitis; CD, Celiac disease; DLE, Discoid lupus erythematosus; GH, Graves' hyperthyroidism; HH, Hashimoto's hypothyroidism; ITP, Immune thrombocytopenic purpura; LS, Localized scleroderma; MN, primary autoimmune membranous nephritis; MS, Multiple sclerosis; PBC, Primary biliary cholangitis; Pso, Psoriasis; T1D, Type 1 diabetes; UC, Ulcerative colitis. **Cancers**: ADGL, adrenal gland cancer; BLRY, cholangiosarcoma, gallbladder & biliary tract cancer; CNS, central nervous system cancer; COLON, colon cancer; KDNY, kidney cancer; LIVER, liver carcinoma, hepatoblastoma, & sarcoma; MYLD, myeloid leukemia (acute and chronic) & multiple myeloma; PANC, pancreatic cancer; SMINT, small intestine cancer; SKM, skin melanoma; STOM, stomach cancer; THYRD, thyroid cancer.

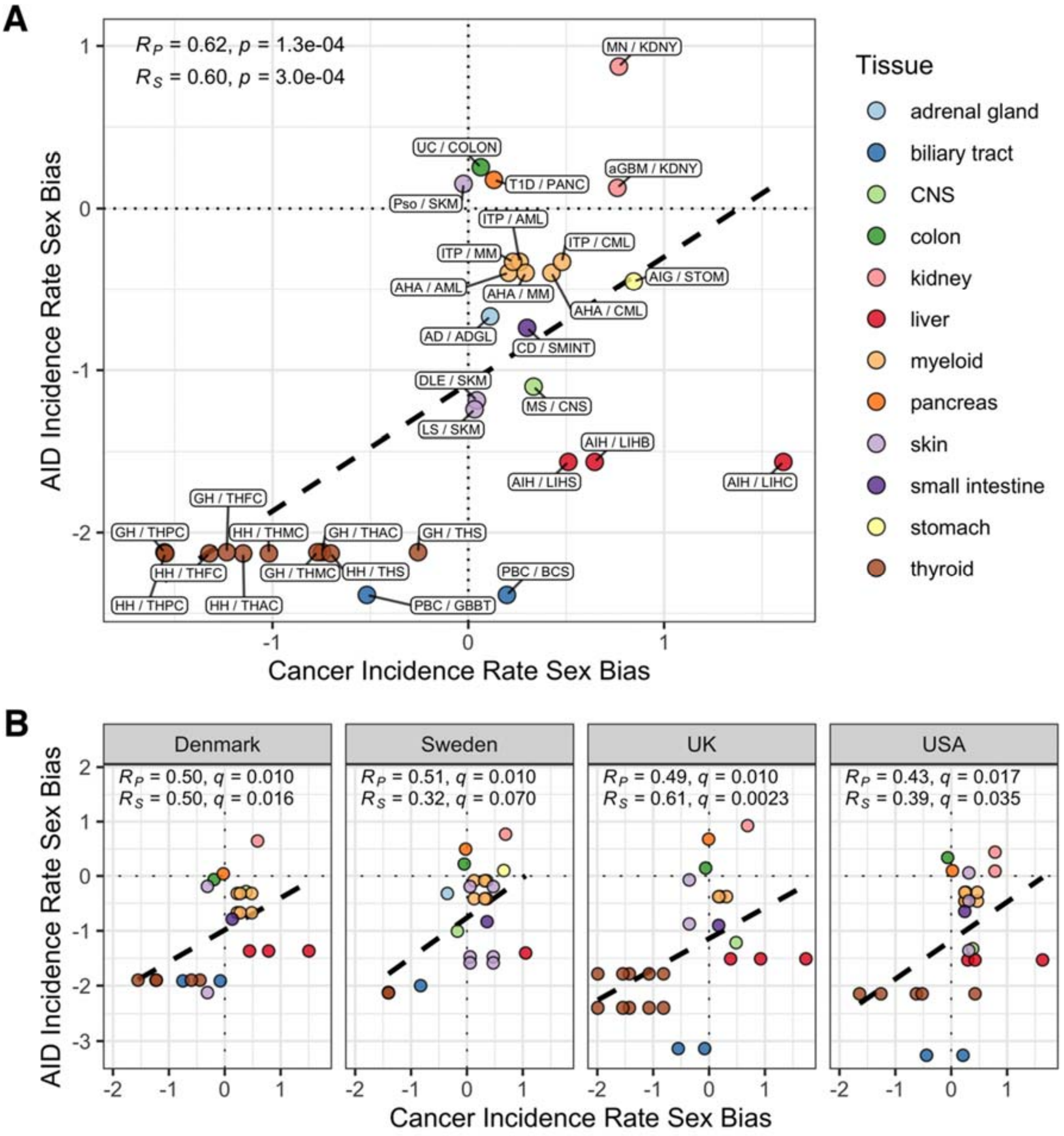

Figure S2. The correlation between incidence rate sex biases for cancers and AIDs (using high-resolution cancer classification). High-resolution cancer classification divides cancers into 21 types. Tissue-matched incidence rate sex biases for cancers (X-axis) and for autoimmune diseases (Y-axis) are displayed across different tissues of origin (circle color indicates the tissue). Positive values in each of the axes indicate male bias; negative values indicate female bias. The dashed line is the linear regression line. Statistics in the top left corner include Pearson's product-moment correlation r-value (R_p) and a t-test p-value (2-sided for A, 1-sided for B); and the Spearman's rank correlation coefficient value (R_s) and a t-test p-value (2-sided for A, 1-sided for

B). For country-level tests, p-values were corrected for multiple testing using the Benjamini-Hochberg method to produce q-values. (**A**) Across-population averages, with the cancer-AID pairs labeled. (**B**) Population-level data for the 4 countries with the largest numbers of data pairs (at least 23 out of 32 cancer-AID pairs), maintaining the tissue color labels used in the top panel (Sweden, 23 pairs; UK, 25 pairs; USA, 25 pairs; Denmark, 23 pairs). **AIDs**: AD, Addison's disease; aGBM, anti-glomerular basement membrane nephritis; AHA, Autoimmune hemolytic anemia; AIG, Autoimmune gastritis; AIH, Autoimmune hepatitis; CD, Celiac disease; DLE, Discoid lupus erythematosus; GH, Graves' hyperthyroidism; HH, Hashimoto's hypothyroidism; ITP, Immune thrombocytopenic purpura; LS, Localized scleroderma; MN, primary autoimmune membranous nephritis; MS, Multiple sclerosis; PBC, Primary biliary cholangitis; Pso, Psoriasis; T1D, Type 1 diabetes; UC, Ulcerative colitis. **Cancers**: ADGL, adrenal gland cancer; AML, acute myeloid leukemia; BCS, liver (biliary) cholangiosarcoma; GBBT, gallbladder & biliary tract cancer; CML, chronic myeloid leukemia; CNS, central nervous system cancer; COLON, colon cancer; KDNY, kidney cancer; LIHC, liver hepatocellular carcinoma; LIHB, liver hepatoblastoma; LIHS, liver hemangiosarcoma; MM, multiple myeloma; PANC, pancreatic cancer; SMINT, small intestine cancer; SKM, skin melanoma; STOM, stomach cancer; THAC, thyroid anaplastic carcinoma; THFC, thyroid follicular carcinoma; THMC, thyroid medullary carcinoma; THPC, thyroid papillary carcinoma; THS, thyroid sarcoma.

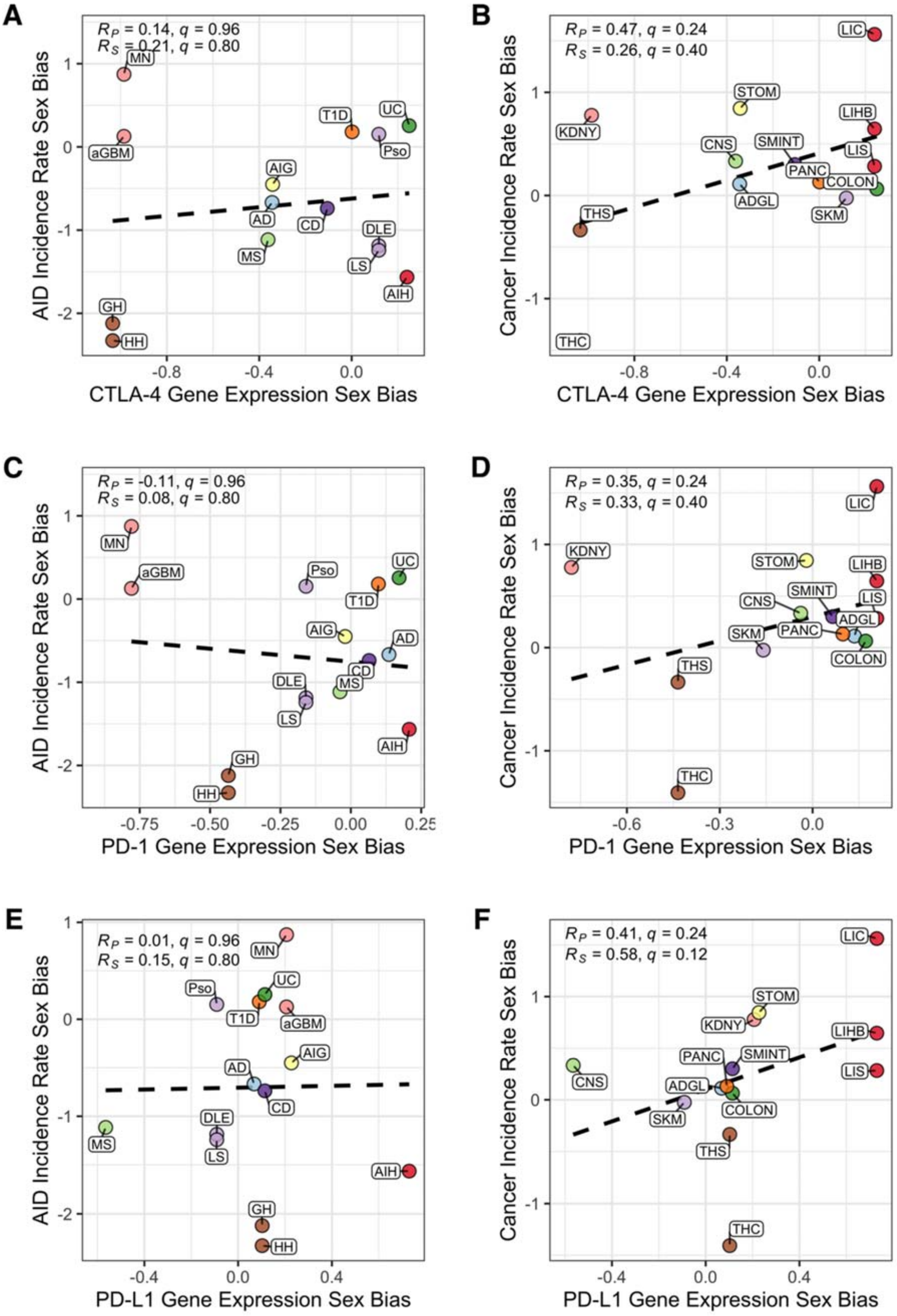
A
R_P = 0.14, q = 0.96
R_S = 0.21, q = 0.80
AID Incidence Rate Sex Bias
CTLA-4 Gene Expression Sex Bias
MN
aGBM
T1D
UC
Pso
AIG
AD
CD
DLE
MS
LS
AIH
GH
HH
B
R_P = 0.47, q = 0.24
R_S = 0.26, q = 0.40
Cancer Incidence Rate Sex Bias
CTLA-4 Gene Expression Sex Bias
LIC
STOM
KDNY
LIHB
CNS
SMINT
LIS
PANC
COLON
ADGL
THS
SKM
THC
C
R_P = -0.11, q = 0.96
R_S = 0.08, q = 0.80
AID Incidence Rate Sex Bias
PD-1 Gene Expression Sex Bias
D
R_P = 0.35, q = 0.24
R_S = 0.33, q = 0.40
Cancer Incidence Rate Sex Bias
PD-1 Gene Expression Sex Bias
E
R_P = 0.01, q = 0.96
R_S = 0.15, q = 0.80
AID Incidence Rate Sex Bias
PD-L1 Gene Expression Sex Bias
F
R_P = 0.41, q = 0.24
R_S = 0.58, q = 0.12
Cancer Incidence Rate Sex Bias
PD-L1 Gene Expression Sex Bias

Figure S3. The correlation between checkpoint gene expression sex bias versus AID and cancer incidence rate sex bias. Gene expression sex bias (X-axis) is calculated as $\log2(TPM_{MALE}/TPM_{FEMALE})$ where TPM is the transcripts-per-million for the gene. AID incidence rate sex bias and cancer incidence rate sex bias (Y-axes) are calculated as $\log2(rate_{MALE}/rate_{FEMALE})$ where $rate_{SEX}$ is the absolute or relative sex-specific incidence rate. For both vertical and horizontal axes, positive sex bias indicates higher value in males and negative sex bias indicates higher value in females. Statistics given in the plot include the Pearson's correlation (R_P) with 2-sided t-test q-value and the Spearman's correlation coefficient (R_S) with 2-sided t-test q-value. P-values were corrected for multiple testing using the Benjamini-Hochberg method to produce q-values. (**A**,**B**) CTLA-4 gene expression sex bias versus (**A**) AID incidence rate sex bias and (**B**) cancer incidence rate sex bias. (**C**,**D**) PD-1 gene expression sex bias versus (**C**) AID incidence rate sex bias and (**D**) cancer incidence rate sex bias. (**E**,**F**) PD-L1 gene expression sex bias versus (**E**) AID incidence rate sex bias and (**F**) cancer incidence rate sex bias. **AIDs**: AD, Addison's disease; aGBM, anti-glomerular basement membrane nephritis; AIG, Autoimmune gastritis; AIH, Autoimmune hepatitis; CD, Celiac disease; DLE, Discoid lupus erythematosus; GH, Graves' hyperthyroidism; HH, Hashimoto's hypothyroidism; LS, Localized scleroderma; MN, primary autoimmune membranous nephritis; MS, Multiple sclerosis; Pso, Psoriasis; T1D, Type 1 diabetes; UC, Ulcerative colitis. **Cancers**: ADGL, adrenal gland cancer; COLON, colon cancer; CNS, central nervous system cancer; KDNY, kidney cancer; LIC, liver carcinoma; LIHB, liver hepatoblastoma; LIS, liver sarcoma; PANC, pancreatic cancer; SKM, melanoma of skin; SMINT, small intestine cancer; STOM, stomach cancer; THC, thyroid carcinoma; THS, thyroid sarcoma.

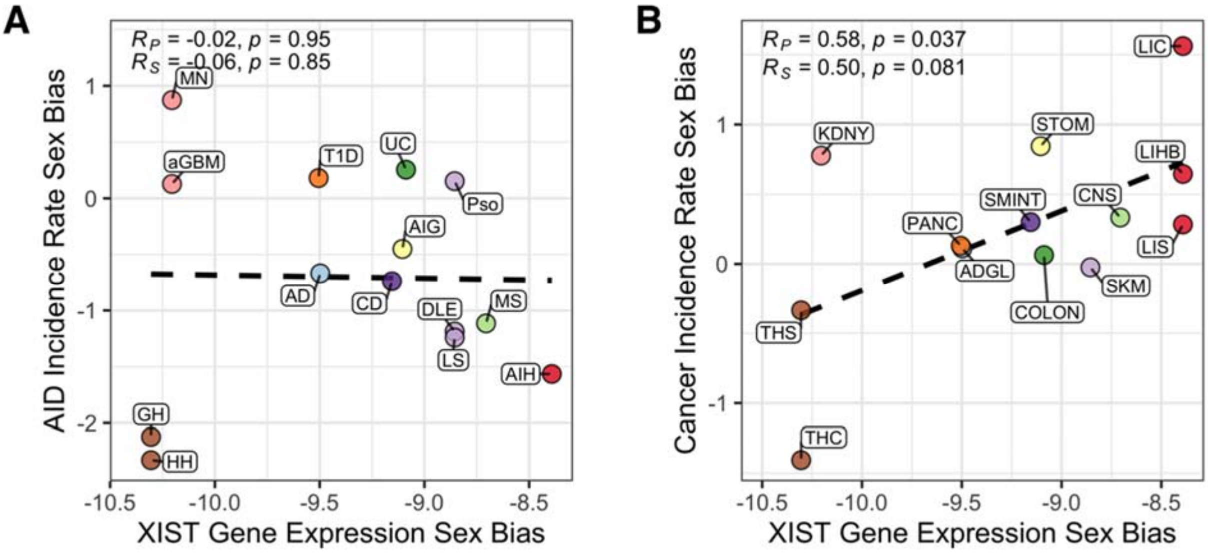

Figure S4. The correlation between XIST gene expression sex bias versus AID and cancer incidence rate sex bias. XIST gene expression sex bias (X-axis) is calculated as log2(TPM_{MALE}/TPM_{FEMALE}) where TPM is the transcripts-per-million for the gene. AID incidence rate sex bias and cancer incidence rate sex bias (Y-axes) are calculated as log2($rate_{MALE}/rate_{FEMALE}$) where $rate_{SEX}$ is the absolute or relative sex-specific incidence rate. For both vertical and horizontal axes, positive sex bias indicates higher value in males and negative sex bias indicates higher value in females. Statistics given in the plot include the Pearson's correlation (R_P) with 1-sided t-test p-value and the Spearman's correlation coefficient (R_S) with 1-sided t-test p-value. (**A**) XIST gene expression sex bias versus AID incidence rate sex bias. (**B**) XIST gene expression sex bias versus cancer incidence rate sex bias. **AIDs**: AD, Addison's disease; aGBM, anti-glomerular basement membrane nephritis; AIG, Autoimmune gastritis; AIH, Autoimmune hepatitis; CD, Celiac disease; DLE, Discoid lupus erythematosus; GH, Graves' hyperthyroidism; HH, Hashimoto's hypothyroidism; LS, Localized scleroderma; MN, primary autoimmune membranous nephritis; MS, Multiple sclerosis; Pso, Psoriasis; T1D, Type 1 diabetes; UC, Ulcerative colitis. **Cancers**: ADGL, adrenal gland cancer; COLON, colon cancer; CNS, central nervous system cancer; KDNY, kidney cancer; LIC, liver carcinoma; LIHB, liver hepatoblastoma; LIS, liver sarcoma; PANC, pancreatic cancer; SKM, melanoma of skin; SMINT, small intestine cancer; STOM, stomach cancer; THC, thyroid carcinoma; THS, thyroid sarcoma.

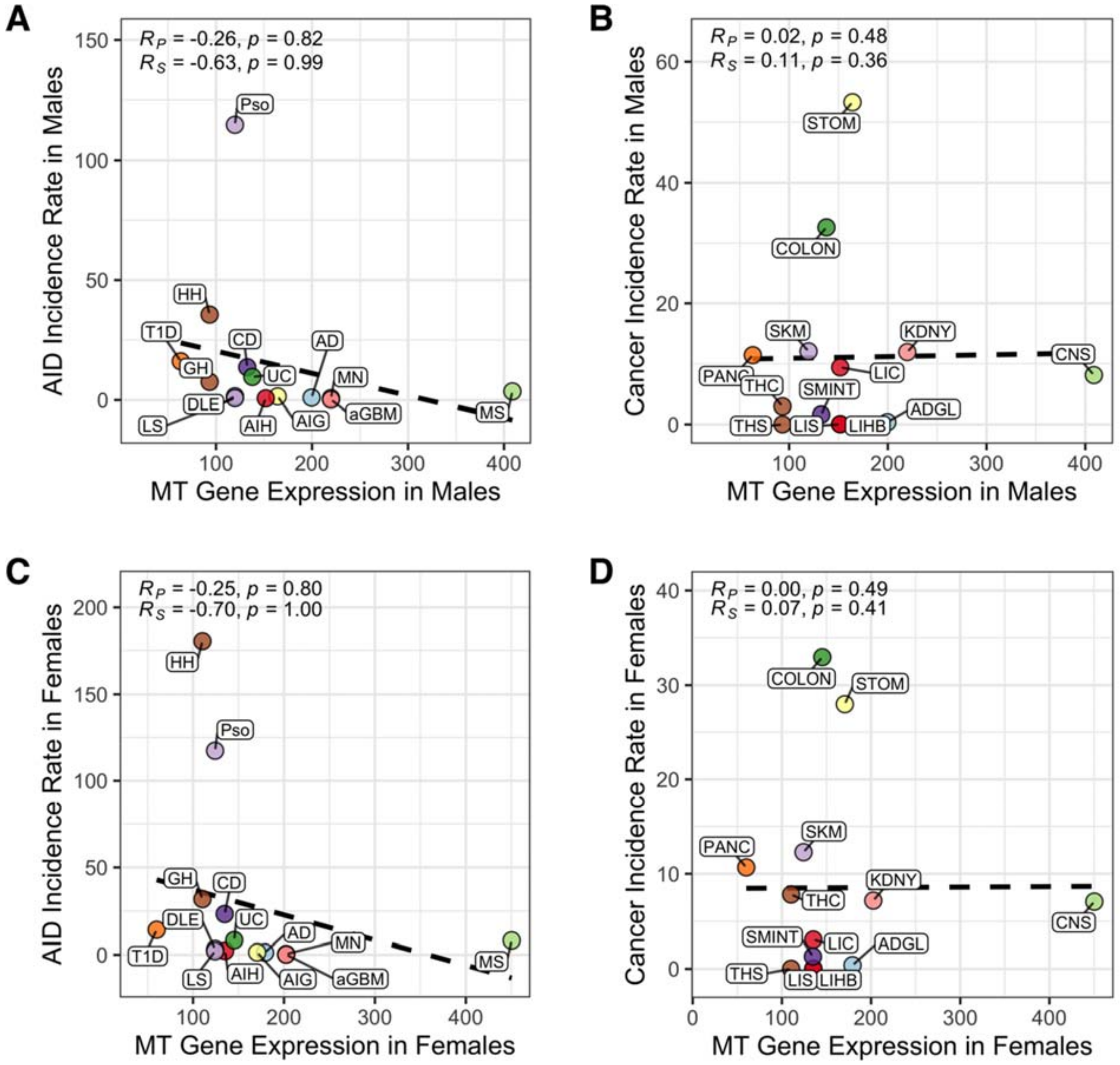

Figure S5. The correlation between mitochondrial gene expression versus AID and cancer incidence rates in males and females. Mitochondrial gene expression (X-axis) is calculated as the geometric mean of expression (in transcripts-per-million, TPM) for mitochondrial genes. AID or cancer incidence rate (Y-axis) is the absolute incidence rate, calculated as cases per 10^5 persons per year. Statistics given in the plot include the Pearson's correlation (R_P) with 1-sided t-test p-value and the Spearman's correlation coefficient (R_S) with 1-sided t-test p-value. (**A**,**B**) Mitochondrial gene expression in males versus (**A**) AID incidence rate in males and (**B**) cancer incidence rate in males. (**C**,**D**) Mitochondrial gene expression in females versus (**C**) AID incidence rate in females and (**D**) cancer incidence rate in females. **AIDs**: AD, Addison's disease; aGBM,

anti-glomerular basement membrane nephritis; AIG, Autoimmune gastritis; AIH, Autoimmune hepatitis; CD, Celiac disease; DLE, Discoid lupus erythematosus; GH, Graves' hyperthyroidism; HH, Hashimoto's hypothyroidism; LS, Localized scleroderma; MN, primary autoimmune membranous nephritis; MS, Multiple sclerosis; Pso, Psoriasis; T1D, Type 1 diabetes; UC, Ulcerative colitis. **Cancers**: ADGL, adrenal gland cancer; COLON, colon cancer; CNS, central nervous system cancer; KDNY, kidney cancer; LIC, liver carcinoma; LIHB, liver hepatoblastoma; LIS, liver sarcoma; PANC, pancreatic cancer; SKM, melanoma of skin; SMINT, small intestine cancer; STOM, stomach cancer; THC, thyroid carcinoma; THS, thyroid sarcoma.
