# Supplementary material for: Sex Biases in Cancer and Autoimmune Disease Incidence Are Strongly Positively Correlated with Mitochondrial Gene Expression across Human Tissues
Source: Cancers (Basel). 2022 Nov 29;14(23):5885. doi: 10.3390/cancers14235885 (PMC9736300; doi:10.3390/cancers14235885)
Supplement: Supplementary file 1 [file cancers-14-05885-s001.zip › cancers-1948266 Supplementary refs.pdf]

Supplementary References for "Sex biases in cancer and autoimmune disease incidence are strongly positively correlated with mitochondrial gene expression across human tissues"

David R. Crawford[a,b], Sanju Sinha[a], Nishanth Ulhas Nair[a], Bríd M. Ryan[c], Jill S. Barnholtz-Sloan[d,e], Stephen M. Mount[b], Ayelet Erez[f], Ken Aldape[g], Philip E. Castle[e,h], Padma S. Rajagopal[a,i], Chi-Ping Day[j], Alejandro A. Schäffer[a] & Eytan Ruppin[a,*]

[a] Cancer Data Science Laboratory, Center for Cancer Research, National Cancer Institute, National Institutes of Health, Bethesda, MD

[b] Department of Cell Biology and Molecular Genetics, University of Maryland, College Park, MD

[c] Laboratory of Human Carcinogenesis, Center for Cancer Research, National Cancer Institute, National Institutes of Health, Bethesda, MD

[d] Center for Biomedical Informatics and Information Technology, National Cancer Institute, National Institutes of Health, Bethesda, MD

[e] Trans-Divisional Research Program, Division of Cancer Epidemiology and Genetics, National Cancer Institute, National Institutes of Health, Bethesda, MD

[f] Department of Biological Regulation, Weizmann Institute of Science, Rehovot, Israel

[g] Laboratory of Pathology, Center for Cancer Research, National Cancer Institute, National Institutes of Health, Bethesda, MD

[h] Division of Cancer Prevention, National Cancer Institute, National Institutes of Health, Bethesda, MD

[i] Women's Malignancies Branch, Center for Cancer Research, National Cancer Institute, National Institutes of Health, Bethesda, MD

[j] Laboratory of Cancer Biology and Genetics, Center for Cancer Research, National Cancer Institute, National Institutes of Health, Bethesda, MD

[*]Corresponding author, eytan.ruppin@nih.gov

Supplementary References for Tables S1, S5-6

Abrahamson, P.E., Hall, S.A., Feudjo-Tepie, M., Mitrani-Gold, F.S., Logie, J. (2009) The incidence of idiopathic thrombocytopenic purpura among adults: a population-based study and literature review. *European Journal of Haematology* 83: 83-89. https://doi.org/10.1111/j.1600-0609.2009.01247.x

Achiron, A., Garty, B.-Z., Menascu, S., Magalashvili, D., Dolev, M., Ben-Zeev, B., Pinhas-Hamiel, O. (2012) Multiple Sclerosis in Israeli Children: Incidence, and Clinical, Cerebrospinal Fluid and Magnetic Resonance Imaging Findings. *Israel Medican Association Journal* 14: 234-239.

Alonso, A., Jick, S.S., Olek, M.J., Hernán, M.A. (2007) Incidence of multiple sclerosis in the United Kingdom: Findings from a population-based cohort. *Journal of Neurology* 254: 1736-1741. https://doi.org/10.1007/s00415-007-0602-z

Alroughani, R., Ahmed, S. F., Behbahani, R., Khan, R., Thussu, A., Alexander, K. J., Ashkanani, A., Nagarajan, V., Al-Hashel, J. (2014) Increasing prevalence and incidence rates of multiple sclerosis in Kuwait. *Multiple Sclerosis Journal* 20(5): 543-547. https://doi.org/10.1177/1352458513504328

Antico-Arciuch, V. G., Dima, M., Liao, X. H., Refetoff, S., Di Cristofano, A. (2010) Cross-talk between PI3K and estrogen in the mouse thyroid predisposes to the development of follicular carcinomas with a higher incidence in females. *Oncogene* 29: 5678-5686. https://doi.org/10.1038/onc.2010.308

Bábíčková, J., Tóthová, L., Lengyelová, E., et al. (2015) Sex Differences in Experimentally Induced Colitis in Mice: a Role for Estrogens. *Inflammation* 38: 1996-2006. https://doi.org/10.1007/s10753-015-0180-7

Baldursdottir, T. R., Bergmann, O. M., Jonasson, J. G., Ludviksson, B. R., Axelsson, T. A., Björnsson, E. S. (2012) The epidemiology and natural history of primary biliary cirrhosis: a nationwide population-based study. *European Journal of Gastroenterology & Hepatology* 24: 824-830. https://doi.org/10.1097/MEG.0b013e328353753d

Bell, L. M., Sedlack, R., Beard, C. M., Perry, H. O., Michet, C. J., Kurland, L. T. (1991) Incidence of Psoriasis in Rochester, Minn, 1980-1983. *Archives of Dermatology* 127(8):1184-1187. https://doi.org/10.1001/archderm.1991.01680070084010

Benjaminsen, E., Olavsen, J., Karlberg, M., Alstadhaug, K.B. (2014) Multiple sclerosis in the far north - incidence and prevalence in Nordland County, Norway, 1970-2010. *BMC Neurology* 14: 226. https://doi.org/10.1186/s12883-014-0226-8

Beretta-Piccoli, B. T., Stirnimann, G., Cerny, A., Semela, D., Hessler, R., Helbling, B., Stickel, F., Kalid-de Bakker, C., Bihl, F., Giostra, E., Sinnreich, M. F., Oneta, C., Baserga, A., Invernizzi, P., Carbone, M., Mertens, J. (2018) Geoepidemiology of Primary Biliary Cholangitis: Lessons from Switzerland. *Clinical Reviews in Allergy & Immunology* 54: 295-306. https://doi.org/10.1007/s12016-017-8656-x

Berglund, J., Christensen, S. B., Hallengren, B. (1990) Total and age-specific incidence of Graves' thyrotoxicosis, toxic nodular goitre and solitary toxic adenoma in Malmö 1970-74. *Journal of Internal Medicine* 227(2): 137-141. https://doi.org/10.1111/j.1365-2796.1990.tb00132.x

Berglund, J., Ericsson, U.-B., Hallengren, B. (1996) Increased incidence of thyrotoxicosis in Malmö during the years 1988-1990 as compared to the years 1970-1974. *Journal of Internal Medicine* 239(1): 57-62. https://doi.org/10.1046/j.1365-2796.1996.415757000.x

Berhan, Y., Waernbaum, I., Lind, T., Möllsten, A., Dahlquist, G. (2011) Thirty Years of Prospective Nationwide Incidence of Childhood Type 1 Diabetes: The Accelerating Increase by Time Tends to Level Off in Sweden. *Diabetes* 60: 577-581. https://doi.org/10.2337/db10-0813

Bertram, J. S., Craig, A. W. (1972) Specific induction of bladder cancer in mice by butyl-(4-hydroxybutyl)-nitrosamine and the effects of hormonal modifications on the sex difference in response. *European Journal of Cancer* 8: 587-594. https://doi.org/10.1016/0014-2964(72)90137-5

Blohmé, G., Nyström, L., Arnqvist, H.J., Lithner, F., Littorin, B., Olsson, P.O., Scherstén, B., Wibell, L., Östman, J. (1992) Male predominance of Type 1 (insulin-dependent) diabetes mellitus in young adults: results from a 5-year prospective nationwide study of the 15-34-year age group in Sweden. *Diabetologia* 35: 56-62. https://doi.org/10.1007/BF00400852

Boberg, K.M., Aadland, E., Jahnsen, J., Raknerud, N., Stiris, M., Bell, H. (1998) Incidence and Prevalence of Primary Biliary Cirrhosis, Primary Sclerosing Cholangitis, and Autoimmune Hepatitis in a Norwegian Population. *Scandinavian Journal of Gastroenterology* 33(1): 99-103. https://doi.org/10.1080/00365529850166284

Bodé, S., Gudmand-høyer, E. (1996) Incidence and Prevalence of Adult Coeliac Disease within a Defined Geographic Area in Denmark. *Scandinavian Journal of Gastroenterology* 31: 694-699. https://doi.org/10.3109/00365529609009152

Boonstra, K., Kunst, A. E., Stadhouders, P. H., Tuynman, H. A., Poen, A. C., van Nieuwkerk, K. M. J., Witteman, E. M., Hamann, D., Witteman, B. J., Beuers, U., Ponsioen, C. Y. (2014) Rising incidence and prevalence of primary biliary cirrhosis: a large population-based study. *Liver International* 34(6): e31-e38. https://doi.org/10.1111/liv.12434

Brola, W., Sobolewski, P. Flaga, S., Fudala, M., Szczuchniak, W., Stoiński, J., Rosolowska, A., Wójcik, J., Kapica-Topczewska, K., Ryglewicz, D. (2016) Prevalence and incidence of multiple sclerosis in central Poland, 2010-2014. *BMC Neurology* 16: 134. https://doi.org/10.1186/s12883-016-0662-8

Bruno, G., Runzo, C., Cavallo-Perin, P., Merletti, F., Rivetti, M., Pinach, S., Novelli, G., Trovati, M., Cerutti, F., Pagano, G. (2005) Incidence of Type 1 and Type 2 Diabetes in Adults Aged 30-49 Years. *Diabetes Care* 28(11): 2613-2619. https://doi.org/10.2337/diacare.28.11.2613

Burger, J. P. W., Roovers, E. A., Drenth, J. P. H., Meijer, J. W. R., Wahab, P. J. (2014) Rising incidence of celiac disease in the Netherlands; an analysis of temporal trends from 1995-2010. *Scandinavian Journal of Gastroenterology* 49(8): 933-941. https://doi.org/10.3109/00365521.2014.915054

Canney, M., O'Hara, P., McEvoy, C., Medani, S., Connaughton, D., Abdalla, A., Doyle, R., Stack, A., O'Seaghdha, C., Clarkson, M., Griffin, M., Holian, J., Dorman, A., Niland, A., Keogan, M., Wallace, E., Conlon, N., Walsh, C., Kelly, A., Little, M. (2016) Spatial and Temporal Clustering of Anti-Glomerular Basement Membrane Disease. *Clinical Journal of the American Society of Nephrology* 11: 1392-1399. https://doi.org/10.2215/CJN.13591215

Carlé, A., Laurberg, P., Pedersen, I. B., Knudsen, N., Perrild, H., Ovesen, L., Rasmussen, L. B., Jorgensen, T. (2006) Epidemiology of subtypes of hypothyroidism in Denmark. *European Journal of Endocrinology* 154: 21-28. https://doi.org/10.1530/eje.1.02068

Chen, Y.-K., Lin, C.-L., Cheng, F.T.-F., Sung, F.-C., Kao, C.-H. (2013) Cancer risk in patients with Hashimoto's thyroiditis: a nationwide cohort study. *British Journal of Cancer* 109: 2496-2501. https://doi.org/10.1038/bjc.2013.597

Cheung, K.-S., Seto, W.-K., Fung, J., Lai, C.-L., Yuen, M.-F. (2017) Epidemiology and Natural History of Primary Biliary Cholangitis in the Chinese: A Territory-Based Study in Hong

Kong between 2000 and 2015. *Clinical and Translational Gastroenterology* 8: e116. https://doi.org/10.1038/ctg.2017.43

Chobot, A., Polanska, J., Brandt, A., Deja, G., Glowinska-Olszewska, B., Pilecki, O., Szadkowska, A., Mysliwiec, M., Jarosz-Chobot, P. (2017) Updated 24-year trend of Type 1 diabetes incidence in children in Poland reveals a sinusoidal pattern and sustained increase. *Diabetic Medicine* 34: 1252-1258. https://doi.org/10.1111/dme.13345

Choi, I.J., Jeong, H.J., Han, D.S., Lee, J.S., Choi, K.H., Kang, S.W., Ha, S.K., Lee, H.Y., Kim, P.K. (2001) An Analysis of 4,514 Cases of Renal Biopsy in Korea. *Yonsei Medical Journal* 42(2): 242-254. https://doi.org/10.3349/ymj.2001.42.2.247

Cilleruelo, M. L., Roman-Reichmann, E., Sanchez-Valverde, F., Donat, E., Manuel-Ramos, J., Martín-Orte, E., López, M.-J., García-Novo, D., García, S., Pavón, P., Martín, M., Ortigosa, L., Barrio, J., Gutierrez, C., Espìn, B., Castillejo, G., Peña-Quintana, L., Hualde, I., Sebastían, M., Calvo, C., Fernández, S., De Manueles, J., Armas, H., Urruzuno-Tellerias, P., Juste, M., Bousoño, C., Ribes-Koninckx, C. (2014) Spanish National Registry of Celiac Disease: Incidence and Clinical Presentation. *Journal of Pediatric Gastroenterology and Nutrition* 59(4): 522-526. https://doi.org/10.1097/MPG.0000000000000446

Cook, L. C., Hillhouse, A. E., Myles, M. H., et al. (2014) The role of estrogen signaling in a mouse model of inflammatory bowel disease: a Helicobacter hepaticus model. *PLoS One* 9: e94209. https://doi.org/10.1371/journal.pone.0094209

Dahlquist, G.G., Nyström, L., Patterson, C.C. (2011) Incidence of Type 1 Diabetes in Sweden Among Individuals Aged 0-34 Years, 1983-2007. *Diabetes Care* 24: 1754-1759. https://doi.org/10.2337/dc11-0056

Daltrozzo, T., Hapfelmeier, A., Donnachie, E., Schneider, A., Hemmer, B. (2018) A Systematic Assessment of Prevalence, Incidence and Regional Distribution of Multiple Sclerosis in Bavaria From 2006 to 2015. *Frontiers in Neurology* 9: 871. https://doi.org/10.3389/fneur.2018.00871

Deguchi, J., Miyamoto, M., Okada, S. (1995) Sex hormone-dependent renal cell carcinogenesis induced by ferric nitrilotriacetate in Wistar rats. *Japanese Journal of Cancer Research* 86: 1068-1071. https://doi.org/10.1111/j.1349-7006.1995.tb03022.x

Dehelean, C. A., Soica, C., Pinzaru, I., et al. (2016) Sex differences and pathology status correlated to the toxicity of some common carcinogens in experimental skin carcinoma. *Food and Chemical Toxicology* 95: 149-158. https://doi.org/10.1016/j.fct.2016.07.007

Delgado, J.-S., Vodonos, A., Delgado, B., Jotkowitz, A., Rosenthal, A., Fich, A., Novack, V. (2012) Primary biliary cirrhosis in Southern Israel: A 20 year follow up study. *European Journal of Internal Medicine* 23: e193-e198. https://doi.org/10.1016/j.ejim.2012.09.004

Delgado, J.-S., Vodonos, A., Malnick, S., Kriger, O., Wilkof-Segev, R., Delgado, B., Novack, V., Rosenthal, A., Menachem, Y., Melzer, E., Fich, A. (2013) Autoimmune hepatitis in southern Israel: A 15-year multicenter study. *Journal of Digestive Diseases* 14: 611-618. https://doi.org/10.1111/1751-2980.12085

de Sá, J., Alcalde-Cabero, E., Almazán-Isla, J., García-López, F., de Pedro-Cuesta, J. (2014) Incidence of multiple sclerosis in Northern Lisbon, Portugal: 1998-2007. *BMC Neurology* 14: 249. https://doi.org/10.1186/s12883-014-0249-1

Durosaro, O., Davis, M.D.P., Reed, K.B., Rohlinger, A.L. (2009) Incidence of Cutaneous Lupus Erythematosus, 1965-2005: A Population-Based Study. *Archives of Dermatology* 145(3): 249-253. https://doi.org/10.1001/archdermatol.2009.21

Eder, L., Widdifield, J., Rosen, C. F., Cook, R., Lee, K.-A., Alhusayen, R., Paterson, M. J., Cheng, S. Y., Jabbari, S., Campbell, W., Bernatsky, S., Gladman, D. D., Tu, K. (2019) Trends in the Prevalence and Incidence of Psoriasis and Psoriatic Arthritis in Ontario, Canada: A Population-Based Study. *Arthritis Care & Research* 71(8): 1084-1091. https://doi.org/10.1002/acr.23743

Egeberg, A., Skov, L., Gislason, G. H., Thyssen, J. P., Mallbris, L. (2017) Incidence and Prevalence of Psoriasis in Denmark. *Acta Dermato-Venereologica* 97: 808-812. https://doi.org/10.2340/00015555-2672

Elhami, S.-R., Mohammad, K., Sahraian, M. A., Eftekhar, H. (2011) A 20-Year Incidence Trend (1989-2008) and Point Prevalence (March 20, 2009) of Multiple Sclerosis in Tehran, Iran: A Population-Based Study. *Neuroepidemiology* 36: 141-147. https://doi.org/10.1159/000324708

Eskandarieh, S., Heydarpour, P., Elhami, S.-R., Sahraian, M. A. (2017) Prevalence and Incidence of Multiple Sclerosis in Tehran, Iran. *Iranian Journal of Public Health* 46(5): 699-704. PMID: 28560202

Fasbender, P., Kölmel, H. (2008) Incidence of Multiple Sclerosis in the Urban Area of Erfurt, Thuringia, Germany. *Neuroepidemiology* 30: 147-151. https://doi.org/10.1159/000122331

Flynn, R.W.V., MacDonald, T.M., Morris, A.D., Jung, R.T., Leese, G.P. (2004) The Thyroid Epidemiology, Audit, and Research Study: Thyroid Dysfunction in the General Population. *The Journal of Clinical Endocrinology & Metabolism* 89(8): 3879-3884. https://doi.org/10.1210/jc.2003-032089

Follansbee, T., Zhou, Y., Wu, X., et al. (2019) Signs of chronic itch in the mouse imiquimod model of psoriaform dermatitis: sex differences and roles of TRPV1 and TRPA1. *Itch (Phila)* 4: e25. https://doi.org/10.1097/itx.0000000000000025

Fox, J. G., Rogers, A. B., Ihrig, M., et al. (2003) *Helicobacter pylori*-associated gastric cancer in INS-GAS mice is gender specific. *Cancer Research* 63: 942-950. https://cancerres.aacrjournals.org/content/63/5/942.long

Frederiksen, H., Schmidt, K. (1999) The Incidence of Idiopathic Thrombocytopenic Purpura in Adults Increases with Age. *Blood* 94(3): 909-913. https://doi.org/10.1182/blood.V94.3.909.415k02_909_913

Furszyfer, J., Kurland, L. T., McConahey, W. M., Woolner, L. B., Elveback, L. R. (1972) Epidemiologic Aspects of Hashimoto's Thyroiditis and Graves' Disease in Rochester, Minnesota (1935-1967), With Special Reference to Temporal Trends. *Metabolism* 21(3): 197-204. https://doi.org/10.1016/0026-0495(72)90041-8

Goday, A., Castell, C., Tresserras, R., Canela, J., Taberner, J.L., Lloveras, G. (1992) Incidence of Type 1 (insulin-dependent) diabetes mellitus in Catalonia, Spain. *Diabetologia* 35: 267-271. https://doi.org/10.1007/BF00400928

González, E.L.M., Johansson, S., Wallander, M.-A., Rodríguez, L.A.G. (2009) Trends in the prevalence and incidence of diabetes in the UK: 1996-2005. *Journal of Epidemiology & Community Health* 63: 332-336. https://doi.org/10.1136/jech.2008.080382

Granieri, E., Malagù, S., Casetta, I., Tola, M. R., Govoni, V., Paolino, E., Monetti, V. C. (1996) Multiple Sclerosis in Italy: A Reappraisal of Incidence and Prevalence in Ferrara. *Archives of Neurology* 53(8): 793-798. https://doi.org/10.1001/archneur.1996.00550080115019

Grimaldi, L., Palmeri, B., Salemi, G., Giglia, G., D'Amelio, M., Grimaldi, R., Vitello, G., Ragonese, P., Savettieri, G. (2007) High Prevalence and Fast Rising Incidence of Multiple

Sclerosis in Caltanissetta, Sicily, Southern Italy. *Neuroepidemiology* 28: 28-32. https://doi.org/10.1159/000097853

Grode, L., Bech, B. H., Jensen, T. M., Humaidan, P., Agerholm, I. E., Plana-Ripoll, O., Ramlau-Hansen, C. H. (2018) Prevalence, incidence, and autoimmune comorbidities of celiac disease: a nation-wide, population-based study in Denmark from 1977 to 2016. *European Journal of Gastroenterology & Hepatology* 30(1): 83-91. https://doi.org/10.1097/MEG.0000000000000992

Grønbæk, L., Otete, H., Ban, L., Crooks, C., Card, T., Jepsen, P., West, J. (2020) Incidence, prevalence and mortality of autoimmune hepatitis in England 1997-2015. A population-based cohort study. *Liver International* 40: 1634-1644. https://doi.org/10.1111/liv.14480

Grønbæk, L., Vilstrup, H., Jepsen, P. (2014) Autoimmune hepatitis in Denmark: Incidence, prevalence, prognosis, and causes of death. A nationwide registry-based cohort study. *Journal of Hepatology* 60: 612-617. https://doi.org/10.1016/j.jhep.2013.10.020

Grönhagen, C.M., Fored, C.M., Granath, F., Nyberg, F. (2011) Cutaneous lupus erythematosus and the association with systemic lupus erythematosus: a population-based cohort of 1088 patients in Sweden. *British Journal of Dermatology* 164: 1335-1341. https://doi.org/10.1111/j.1365-2133.2011.10272.x

Hanko, J.B., Mullan, R.N., O'Rourke, D.M., McNamee, P.T., Maxwell, A.P., Courtney, A.E. (2009) The changing pattern of adult primary glomerular disease. *Nephrology Dialysis Transplantation* 24(10): 3050-3054. https://doi.org/10.1093/ndt/gfp254

Hansen, D., Möller, S., Andersen, K., Gaist, D., Frederiksen, H. (2020) Increasing Incidence and Prevalence of Acquired Hemolytic Anemias in Denmark, 1980-2016. *Clinical Epidemiology* 12: 497-508. https://doi.org/10.2147/CLEP.S250250

Harjutsalo, V., Sund, R., Knip, M., Groop, P.-H. (2013) Incidence of Type 1 Diabetes in Finland. *Journal of the American Medical Association* 310(4): 427-428. https://doi.org/10.1001/jama.2013.8399

Haug, K., Schrumpf, E., Barstad, S., Fluge, G., Halvorsen, J. (1988) Epidemiology of Ulcerative Colitis in Western Norway. *Scandinavian Journal of Gastroenterology* 23: 517-522. https://doi.org/10.3109/00365528809093904

Haynes, A., Bower, C., Bulsara, M.K., Jones, T.W., Davis, E.A. (2004) Continued increase in the incidence of childhood Type 1 diabetes in a population-based Australian sample (1985-2002). *Diabetologia* 47: 866-870. https://doi.org/10.1007/s00125-004-1385-8

Heaf, J., Løkkegaard, H., Larsen, S. (1999) The epidemiology and prognosis of glomerulonephritis in Denmark 1985-1997. *Nephrology Dialysis Transplantation* 14(8): 1889-1897. https:/doi.org/10.1093/ndt/14.8.1889

Heijmans, J., Wielenga, M. C., Rosekrans, S. L., et al. (2014) Oestrogens promote tumorigenesis in a mouse model for colitis-associated cancer. *Gut* 63: 310-316. https://doi.org/10.1136/gutjnl-2012-304216

Herrick, A. L., Ennis, H., Bhushan, M., Silman, A. J., Baildam, E. M. (2010) Incidence of Childhood Linear Scleroderma and Systemic Sclerosis in the UK and Ireland. *Arthritis Care & Research* 62(2): 213-218. https://doi.org/10.1002/acr.20070

Hussain, Y. S., Hookham, J. C., Allahabadia, A., Balasubramanian, S. P. (2017) Epidemiology, management and outcomes of Graves' disease---real life data. *Endocrine* 56: 568-578. https://doi.org/10.1007/s12020-017-1306-5

Icen, M., Crowson, C. S., McEvoy, M. T., Dann, F. J., Gabriel, S. E., Kremers, H. M. (2009) Trends in incidence of adult-onset psoriasis over three decades: A population-based study. *Journal of the American Academy of Dermatology* 60: 394-401. https://doi.org/10.1016/j.jaad.2008.10.062

Jacobsen, B., Fallingborg, J., Rasmussen, H., Nielsen, K., Drewes, A., Puho, E., Nielsen, G., Sørensen, H. (2006) Increase in incidence and prevalence of inflammatory bowel disease in northern Denmark: a population-based study, 1978-2002. *European Journal of Gastroenterology & Hepatology* 18: 601-606. https://doi.org/10.1097/00042737-200606000-00005

Ji, J., Sundquist, J., Sundquist, K. (2016) Gender-specific incidence of autoimmune diseases from national registers. *Journal of Autoimmunity* 69: 102-106. https://doi.org/10.1016/j.jaut.2016.03.003

Jiménez-Rivera, C., Ling, S. C., Ahmed, N., Yap, J., Aglipay, M., Barrowman, N., Graitson, S., Critch, J., Rashid, M., Ng, V. L., Roberts, E. A., Brill, H., Dowhaniuk, J. K., Bruce, G., Bax, K., Deneau, M, Guttman, O. R., Schreiber, R. A., Martin, S., Alvarez, F. (2015) Incidence

and Characteristics of Autoimmune Hepatitis. *Pediatrics* 136(5): e1237. https://doi.org/10.1542/peds.2015-0578

Kanth, R., Shrestha, R. B., Rai, I., VanWormer, J. J., Roy, P. K. (2017) Incidence of Primary Biliary Cholangitis in a Rural Midwestern Population. *Clinical Medicine & Research* 15(1-2): 13-18. https://doi.org/10.3121/cmr.2017.1351

Kim, W. R., Lindor, K. D., Locke, G. R. III, Therneau, T. M., Homburger, H. A., Batts, K. P., Yawn, B. P., Petz, J. L., Melton, L. J. III, Dickson, E. R. (2000) Epidemiology and Natural History of Primary Biliary Cirrhosis in a U.S. Community. *Gastroenterology* 119: 1631-1636. https://doi.org/10.1053/gast.2000.20197

Kim, K.-A., Ki, M., Choi, H. Y., Kim, B. H., Jang, E. S., Jeong, S.-H. (2016) Population-based epidemiology of primary biliary cirrhosis in South Korea. *Ailmentary Pharmacology and Therapeutics* 43: 154-162. https://doi.org/10.1111/apt.13448

Kim, B. H., Choi, H. Y., Ki, M., Kim, K.-A., Jang, E. S., Jeong, S.-H. (2017) Population-based prevalence, incidence, and disease burden of autoimmune hepatitis in South Korea. *PLoS ONE* 12(8): e0182391. https://doi.org/10.1371/journal.pone.0182391

Kim, E. S., Han, K., Kim, M. K., Park, Y.-M., Baek, K.-H., Moon, S. D., Han, J.-H., Song, K.-H., Kwon, H.-S. (2017) Impact of metabolic status on the incidence of psoriasis: a Korean nationwide cohort study. *Scientific Reports* 7: 1989. https://doi.org/10.1038/s41598-017-01983-y

King, D., Reulen, R., Thomas, T., Chandan, J., Thayakaran, R., Subramanian, A., Gokhale, K., Bhala N., Nirantharakumar, K., Adderley, N., Trudgill, N. (2020) Changing patterns in the epidemiology and outcomes of inflammatory bowel disease in the United Kingdom: 2000-2018. *Alimentary Pharmacology & Therapeutics* 51: 922-934. https://doi.org/10.1111/apt.15701

Kingwell, E., Zhu, F., Marrie, R. A., Fisk, J. D., Wolfson, C., Warren, S., Profetto-McGrath, J., Svenson, L. W., Jette, N., Bhan, V., Yu, B. N., Elliott, L., Tremlett, H. (2015) High incidence and increasing prevalence of multiple sclerosis in British Columbia, Canada: findings from over two decades (1991-2020). *Journal of Neurology* 262: 2352-2363. https://doi.org/10.1007/s00415-015-7842-0

Kinnunen, E. (1984) Multiple sclerosis in Finland: Evidence of increasing frequency and uneven geographic distribution. *Neurology* 34: 457-461. https://doi.org/10.1212/wnl.34.4.457

Kivelä, L., Kaukinen, K., Lähdeaho, M.-L., Huhtala, H., Ashorn, M., Ruuska, T., Hiltunen, P., Visakorpi, J., Mäki, M., Kurppa, K. (2015) Presentation of Celiac Disease in Finnish Children Is No Longer Changing: A 50-Year Perspective. *The Journal of Pediatrics* 167: 1109-1115. https://doi.org/10.1016/j.jpeds.2015.07.057

Klein, N., Ray, P., Carpenter, D., Hansen, J., Lewis, E., Fireman, B., Black, S., Galindo, C., Schmidt, J., Baxter, R. (2010) Rates of autoimmune diseases in Kaiser Permanente for use in vaccine adverse event safety studies. *Vaccine* 28: 1062-1068. https://doi.org/10.1016/j.vaccine.2009.10.115

Koch-Henriksen, N., Hyllested, K. (1988) Epidemiology of multiple sclerosis: incidence and prevalence rates in Denmark 1948-64 based on the Danish Multiple Sclerosis Registry. *Acta Neurologica Scandinavica* 78: 369-380. https://doi.org/10.1111/j.1600-0404.1988.tb03672.x

Koton, S., the IIRSG (2007) Incidence of type 1 diabetes mellitus in the 0- to 17-yr-old Israel population, 1997-2003. *Pedriatic Diabetes* 8(2): 60-66. https://doi.org/10.1111/j.1399-5448.2007.00230.x

Koulentaki, M., Mantaka, A., Sifaki-Pistolla, D., Thalassinos, E., Tzanakis, N., Kouroumalis, E. (2014) Geoepidemiology and space-time analysis of Primary biliary cirrhosis in Crete, Greece. *Liver International* 34: e200-e207. https://doi.org/10.1111/liv.12479

Kramer, M.A., van der Maas, N.A.T., van Soest, E.M., Kemmeren, J.M., de Melker, H.E., Sturkenboom, M.C.J.M. (2012) Incidence of Multiple Sclerosis in the General Population in the Netherlands, 1996-2008. *Neuroepidemiology* 39: 96-102. https://doi.org/10.1159/000338678

Krökki, O., Bloigu, R., Reunanen, M., Remes, A. M. (2011) Increasing incidence of multiple sclerosis in women in Northern Finland. *Multiple Sclerosis Journal* 17(2): 133-138. https://doi.org/10.1177/1352458510384012

Lai, C.-H., Tseng, H.-F. (2009) Population-Based Epidemiological Study of Neurological Diseases in Taiwan: 1. Creutzfeldt-Jakob Disease and Multiple Sclerosis. *Neuroepidemiology* 33: 247-253. https://doi.org/10.1159/000229779

Lamba, M., Ngu, J., Stedman, C. (2021) Trends in Incidence of Autoimmune Liver Diseases and Increasing Incidence of Autoimmune Hepatitis. *Clinical Gastroenterology and Hepatology* 19: 573-579. https://doi.org/10.1016/j.cgh.2020.05.061

Langholz, E., Munkholm, P., Nielsen, O., Kreiner, S., Binder, V. (1991) Incidence and Prevalence of Ulcerative Colitis in Copenhagen County from 1962 to 1987. *Scandinavian Journal of Gastroenterology* 26: 1247-1256. https://doi.org/10.3109/00365529108998621

Lanzini, A., Villanacci, V., Apillan, N., Lanzarotto, F., Pirali, F., Amato, M., Indelicato, A., Scarcella, C., Donato, F. (2005) Epidemiological, clinical and histopathologic characteristics of celiac disease: Results of a case-finding population-based program in an Italian community. *Scandinavian Journal of Gastroenterology* 40: 950-957. https://doi.org/10.1080/00365520510023107

Lechtman, N., Shamir, R., Cohen, S., Chodick, G., Kariv, R., Supino-Rosin, L., Weintraub, Y., Yerushalemy-Feler, A. (2021) Increased incidence of coeliac disease autoimmunity rate in Israel: a 9-year analysis of population-based data. *Alimentary Pharmacology & Therapeutics* 53(6): 696-703. https://doi.org/10.1111/apt.16282

Lee, S. M., Kim, N., Son, H. J., et al. (2016) The Effect of Sex on the Azoxymethane/Dextran Sulfate Sodium-treated Mice Model of Colon Cancer. *Journal of Cancer Prevention* 21: 271-278. https://doi.org/10.15430/JCP.2016.21.4.271

Leese, G.P., Flynn, R.V., Jung, R.T., MacDonald, T.M., Murphy, M.J., Morris, A.D. (2008) Increasing prevalence and incidence of thyroid disease in Tayside, Scotland: the Thyroid Epidemiology Audit and Research Study (TEARS). *Clinical Endocrinology* 68: 311-316. https://doi.org/10.1111/j.1365-2265.2007.03051.x

Lertpiriyapong, K., Whary, M. T., Muthupalani, S., et al. (2014) Gastric colonisation with a restricted commensal microbiota replicates the promotion of neoplastic lesions by diverse intestinal microbiota in the *Helicobacter pylori* INS-GAS mouse model of gastric carcinogenesis. *Gut* 63: 54-63. https://doi.org/10.1136/gutjnl-2013-305178

Levy-Marchal, C., Papoz, L., de Beaufort, C., Doutreix, J., Froment, V., Collignon, A., Garros, B., Schleret, Y., Czernichow, P. (1990) Incidence of juvenile Type 1 (insulin-dependent) diabetes mellitus in France. *Diabetalogia* 33: 465-469. https://doi.org/10.1007/BF00405107

Lleo, A., Jepsen, P., Morenghi, E., Carbone, M., Moroni, L., Battezzati, P. M., Podda, M., Mackay, I. R., Gershwin, M. E., Invernizzi, P. (2016) Evolving Trends in Female to Male Incidence and Male Mortality of Primary Biliary Cholangitis. *Scientific Reports* 6: 25906. https://doi.org/10.1038/srep25906

Loftus, C., Loftus Jr., E., Harmsen, S., Zinsmeister, A., Tremaine, W., Melton III, L., Sandborn, W. (2007) Update on the Incidence and Prevalence of Crohn's Disease and Ulcerative Colitis in Olmsted County, Minnesota, 1940-2000. *Inflammatory Bowel Diseases* 13: 254-261. https://doi.org/10.1002/ibd.20029

Longnecker, D. S., Sumi, C. (1990) Effects of sex steroid hormones on pancreatic cancer in the rat. *International Journal of Pancreatology* 7: 159-165. https://doi.org/10.1007/BF02924233

Lönnbro-Widgren, J., Mölne, J., Haraldsson, B., Nyström, J. (2016) Treatment pattern in patients with idiopathic membranous nephropathy--practices in Sweden at the start of the millennium. *Clinical Kidney Journal* 9(2): 227-233. https://doi.org/10.1093/ckj/sfv152

Ludvigsson, J., Rubio-Tapia, A., van Dyke, C., Melton III, L., Zinsmeister, A., Lahr, B., Murray, J. (2013) Increasing Incidence of Celiac Disease in a North American Population. *The American Journal of Gastroenterology* 108: 818-824. https://doi.org/10.1038/ajg.2013.60

Marschall, H.-U., Henriksson, I., Lindberg, S., Söderdahl, F., Thuresson, M., Wahlin, S., Ludvigsson, J. F. (2019) Incidence, prevalence and outcome of primary biliary cholangitis in a nationwide Swedish population-based cohort. *Scientific Reports* 9: 11525. https://doi.org/10.1038/s41598-019-47890-2

Maté-Jimenez, J., Muñoz, S., Vicent, D., Pajares, J. M. (1994) Incidence and Prevalence of Ulcerative Colitis and Crohn's Disease in Urban and Rural Areas of Spain from 1981 to 1988. *Journal of Clinical Gastroenterology* 18(1): 27-31. https://doi.org/10.1097/00004836-199401000-00008

Mayer-Davis, E.J., Lawrence, J.M., Dabelea, D., Divers, J., Isom, S., Dolan, L., Imperatore, G., Linder, B., Marcovina, S., Pettitt, D.J., Pihoker, C., Saydah, S., Wagenknecht, L. (2017) Incidence Trends of Type 1 and Type 2 Diabetes among Youths, 2002-2012. *The New England Journal of Medicine* 376: 1419-1429. https://doi.org/10.1056/NEJMoa1610187

McCullough, M. L., Wan, N., Pezzolesi, M. G., Collins, T. W., Grineski, S. E., Wei, Y. D., Lazaro-Guevara, J., Frodsham, S. G., Vanderslice, J. A., Holmen, J. R., Srinivas, T. R., Clements, S. A. (2021) Type 1 Diabetes incidence among youth in Utah: A geographical analysis. *Social Science & Medicine* 278: 113952. https://doi.org/10.1016/j.socscimed.2021.113952

McNally, R. J. Q., James, P. W., Ducker, S., Norman, P. D., James, O. F. W. (2014) No Rise in Incidence but Geographical Heterogeneity in the Occurrence of Primary Biliary Cirrhosis in

North East England. *American Journal of Epidemiology* 179(4): 492-498. https://doi.org/10.1093/aje/kwt308

Meyer, G., Neumann, K., Badenhoop, K., Linder, R. (2014) Increasing prevalence of Addison's disease in German females: health insurance data 2008-2012. *European Journal of Endocrinology* 170: 367-373. https://doi.org/10.1530/EJE-13-0756

Molinié, F., Gower-Rousseau, C., Yzet, T., Merle, V., Grandbastien, B., Marti, R., Lerebours, E., Dupas, J-L., Colombel, J-F., Salomez, J-L., Cortot, A. (2004) Opposite evolution in incidence of Crohn's disease and ulcerative colitis in Northern France (1988-1999). *Gut* 53: 843-848. https://doi.org/10.1136/gut.2003.025346

Mostbeck, A., Galvan, G., Bauer, P., Eber, O., Atefie, K., Dam, K., Feichtinger, H., Fritzsche, H., Haydl, H., Köhn, H., König, B., Koriska, K., Kroiss, A., Lind, P., Markt, B., Maschek, W., Pesl, H., Ramschak-Schwarzer, S., Riccabona, G., Stockhammer, M., Zechmann, W. (1998) The incidence of hyperthyroidism in Austria from 1987 to 1995 before and after an increase in salt iodization in 1990. *European Journal of Nuclear Medicine* 25: 367-374. https://doi.org/10.1007/s002590050234

Moulis, G., Palmaro, A., Montastruc, J.-L., Godeau, B., Lapeyre-Mestre, M., Sailler, L. (2014) Epidemiology of incident immune thrombocytopenia: a nationwide population-based study in France. *Blood* 124(22): 3308-3315. https://doi.org/10.1182/blood-2014-05-578336

Moum, B., Vatn, M., Ekbom, A., Aadland, E., Fausa, O., Lygren, I., Sauar, J., Schulz, T., Stray, N. (1996) Incidence of Ulcerative Colitis and Indeterminate Colitis in Four Counties of Southeastern Norway, 1990-93 A Prospective Population-Based Study. *Scandinavian Journal of Gastroenterology* 31: 362-366. https://doi.org/10.3109/00365529609006411

Myers, R. P., Shaheen, A. A. M., Fong, A., Burak, K. W., Wan, A., Swain, M. G., Hilsden, R. J., Sutherland, L., Quan, H. (2009) Epidemiology and Natural History of Primary Biliary Cirrhosis in a Canadian Health Region: A Population-Based Study. *Hepatology* 50(6): 1884-1892. https://doi.org/10.1002/hep.23210

Myszor, M., James, O. F. W. (1990) The Epidemiology of Primary Biliary Cirrhosis in North-east England: an Increasingly Common Disease? *QJM: An International Journal of Medicine* 75(1): 377-385. https://doi.org/10.1093/oxfordjournals.qjmed.a068447

Naugler, W. E., Sakurai, T., Kim, S., et al. (2007) Gender disparity in liver cancer cue to sex differences in MyD88-dependent IL-6 production. *Science* 317: 121-124. https://doi.org/10.1126/science.1140485

Negrato, C. A., Dias, J. P. L., Teixeira, M. F., Dias, A., Salgado, M. H., Lauris, J. R., Montenegro, R. M. Jr., Gomes, M. B., Jovanovic, L. (2010) Temporal trends in incidence of Type 1 diabetes between 1986 and 2006 in Brazil. *Journal of Endocrinological Investigation* 33: 373-377. https://doi.org/10.3275/6439

Neylon, A.J., Saunders, P.W.G., Howard, M.R., Proctor, S.J., Taylor, P.R.A. (2003) Clinically significant newly presenting autoimmune thrombocytopenic purpura in adults: a prospective study of a population-based cohort of 245 patients. *British Journal of Haematology* 122: 966-974. https://doi.org/10.1046/j.1365-2141.2003.04547.x

Ngu, J. H., Gearry, R. B., Frampton, C. M., Stedman C. A. M. (2012) Mortality and the Risk of Malignancy in Autoimmune Liver Diseases: A Population-Based Study in Canterbury, New Zealand. *Hepatology* 55: 522-529. https://doi.org/10.1002/hep.24743

Nomura, K. Demura, H., Saruta, T. (1994) Addison's Disease in Japan: Characteristics and Changes Revealed in a Nationwide Survey. *International Medicine* 33: 602-606. https://doi.org/10.2169/internalmedicine.33.602

Nordenvall, B., Broström, O., Berglund, M., Monsen, U., Nordenström, J., Sörstad, J., Hellers, G. (1985) Incidence of Ulcerative Colitis in Stockholm County 1955-1979. *Scandinavian Journal of Gastroenterology* 20: 783-790. https://doi.org/10.3109/00365528509088823

Nørgård, B. M., Nielsen, J., Fonager, K., Kjeldsen, J., Jacobsen, B. A., Qvist, N. (2014) The incidence of ulcerative colitis (1995-2011) and Crohn's disease (1995-2012) --- Based on nationwide Danish registry data. *Journal of Crohn's and Colitis* 8: 1274-1280. https://doi.org/10.1016/j.crohns.2014.03.006

Nyström, H. F., Jansson, S., Berg, G. (2013) Incidence rate and clinical features of hyperthyroidism in a long-term iodine sufficient area of Sweden (Gothenburg) 2003-2005. *Clinical Endocrinology* 78(5): 768-776. https://doi.org/10.1111/cen.12060

Onda, Y., Sugihara, S., Ogata, T., Yokoya, S., Yokoyama, T., Tajima, N. (2017) Incidence and prevalence of childhood-onset Type 1 diabetes in Japan: the T1D study. *Diabetic Medicine* 34: 909-915. https://doi.org/10.1111/dme.13295

Orton, S.-M., Herrera, B. M., Yee, I. M., Valdar, W., Ramagopalan, S. V., Sadovnick, A. D., Ebers, G. C. (2006) Sex ratio of multiple sclerosis in Canada: a longitudinal study. *Lancet Neurology* 5: 932-936. https://doi.org/10.1016/S1474-4422(06)70581-6

Östman J., Lönnberg G., Arnqvist H.J., Blohmé G., Bolinder J., Ekbom Schnell A., Eriksson J.W., Gudbjörnsdottir S., Sundkvist G., Nyström L. (2008) Gender differences and temporal variation in the incidence of type 1 diabetes: results of 8012 cases in the nationwide Diabetes Incidence Study in Sweden 1983-2002. *Journal of Internal Medicine* 263(4):386-94. https://doi.org/10.1111/j.1365-2796.2007.01896.x

O'Connell, K., Tubridy, N., Hutchinson, M., McGuigan, C. (2017) Incidence of multiple sclerosis in the Republic of Ireland: A prospective population-based study. *Multiple Sclerosis and Related Disorders* 13: 75-80. https://doi.org/10.1016/j.msard.2017.02.010

O'Shaughnessy, M.M., Hogan, S.L., Poulton, C.J., Falk, R.J., Singh, H.K., Nickeleit, V., Jennette, J.C. (2017) Temporal and Demographic Trends in Glomerular Disease Epidemiology in the Southeastern United States, 1986-2015. *Clinical Journal of the American Society of Nephrology* 12: 614-623. https://doi.org/10.2215/CJN.10871016

Otani, Y., Ichii, O., Masum, M. A., Namba, T., Nakamura, T., Kon, Y. (2021) Castrated autoimmune glomerulonephritis mouse model shows attenuated glomerular sclerosis with altered parietal epithelial cell phenotype. *Experimental Biology and Medicine* 246: 1318-1329. https://doi.org/10.1177/1535370221996010

Ott, C., Obermeier, F., Thieler, S., Kemptner, D., Bauer, A., Schölmerich, J., Rogler, G., Timmer, A. (2008) The incidence of inflammatory bowel disease in a rural region of Southern Germany: a prospective population-based study. *European Journal of Gastroenterology & Hepatology* 20: 917-923. https://doi.org/10.1097/MEG.0b013e3282f97b33

Papathanasopoulos, P., Gourzoulidou, E., Messinis, L., Georgiou, V., Leotsinidis, M. (2008) Prevalence and Incidence of Multiple Sclerosis in Western Greece: A 23-Year Survey. *Neuroepidemiology* 30: 167-173. https://doi.org/10.1159/000122334

Parkman, G. L., Kircher, D. A., Stehn, C. M., McMahon, M., Holmen, S. L. (2021) Model-dependent outcomes: Sex as a biological variable in preclinical mouse models of melanoma. *Pigment Cell & Melanoma Research* 34: 655-658. https://doi.org/10.1111/pcmr.12940

Peterson, L.S., Nelson, A.M., Su, W.P., Mason, T., O'Fallon, W.M., Gabriel, S.E. (1997) The epidemiology of morphea (localized scleroderma) in Olmsted County 1960-1993. *The Journal of Rheumatology* 24(1): 73-80. PMID: 9002014.

Petersen, M.P., Möller, S., Bygum, A., Voss, A., Bliddal, M. (2018) Epidemiology of cutaneous lupus erythematosus and the associated risk of systemic lupus erythematosus: a nationwide cohort study in Denmark. *Lupus* 27: 1424-1430. https://doi.org/10.1177/0961203318777103

Piffaretti, C., Mandereau-Bruno, L., Guilmin-Crepon, S., Choleau, C., Coutant, R., Fosse-Edorh, S. (2019) Trends in childhood type 1 diabetes incidence in France, 2010-2015. *Diabetes Research and Clinical Practice* 149: 200-207. https://doi.org/10.1016/j.diabres.2018.11.005

Pla, X., Vergara, M., Gil, M., Dalmau, B., Cisteró, B., Bella, R. M., Real, J. (2007) Incidence, prevalence and clinical course of primary biliary cirrhosis in a Spanish community. *European Journal of Gastroenterology & Hepatology* 19: 859-864. https://doi.org/10.1097/MEG.0b013e328277594a

Pugliatti, M., Riise, T., Sotgiu, M.A., Sotgiu, S., Satta, W.M., Mannu, L., Sanna, G., Rosati, G. (2005) Increasing Incidence of Multiple Sclerosis in the Province of Sassari, Northern Sardinia. *Neuroepidemiology* 25: 129-134. https://doi.org/10.1159/000086677

Puustinen, L., Barner-Rasmussen, N., Pukkala, E., Färkkilä, M. (2019) Incidence, prevalence, and causes of death of patients with autoimmune hepatitis: A nationwide register-based cohort study in Finland. *Digestive and Liver Disease* 51: 1294-1299. https://doi.org/10.1016/j.dld.2019.01.015

Rautiainen, H., Salomaa, V., Niemalä, S., Karvonen, A.-L., Nurmi, H., Isoniemi, H., Färkkilä, M. (2007) Prevalence and incidence of primary biliary cirrhosis are increasing in Finland. *Scandinavian Journal of Gastroenterology* 42(11): 1347-1353. https://doi.org/10.1080/00365520701396034

Remmel, T., Remmel, H., Uibo, R., Salupere, V. (1995) Primary Biliary Cirrhosis in Estonia with Special Reference to Incidence, Prevalence, Clinical Features, and Outcome. *Scandinavian Journal of Gastroenterology* 30(4): 367-371. https://doi.org/10.3109/00365529509093292

Ress, K., Luts, K., Rägo, T., Pisarev, H., Uibo, O. (2012) Nationwide study of childhood celiac disease incidence over a 35-year period in Estonia. *European Journal of Pediatrics* 171: 1823-1828. https://doi.org/10.1007/s00431-012-1835-0

Ribbons, K., Lea, R., Tiedeman, C., Mackenzie, L., Lechner-Scott, J. (2017) Ongoing increase in incidence and prevalence of multiple sclerosis in Newcastle, Australia: A 50-year study. *Multiple Sclerosis Journal* 23(8): 1063-1071. https://doi.org/10.1177/1352458516671819

Riolobos, L., Gad, E. A., Treuting, P. M., et al. (2019) The Effect of Mouse Strain, Sex, and Carcinogen Dose on Toxicity and the Development of Lung Dysplasia and Squamous Cell Carcinomas in Mice. *Cancer Prevention Research* 12: 507-516. https://doi.org/10.1158/1940-6207.CAPR-18-0442

Ríos, M.S., Moy, C.S., Serrano, R.M., Asensio, A.M., de Tomás Labat, M.E., Romero, G.Z., Herrera, J. (1990) Incidence of Type 1 (insulin-dependent) diabetes mellitus in subjects 0-14 years of age in the Comunidad of Madrid, Spain. *Diabetologia* 33: 422-424. https://doi.org/10.1007/BF00404093

Rönnblom, A., Samuelsson, S., Ekbom, A. (2010) Ulcerative colitis in the county of Uppsala 1945-2007: Incidence and clinical characteristics. *Journal of Crohn's and Colitis* 4: 532-536. https://doi.org/10.1016/j.crohns.2010.03.003

Rossini, A. A., Williams, R. M., Appel, M. C., Like, A. A. (1978) Sex differences in the multiple-dose streptozotocin model of diabetes. *Endocrinology* 103: 1518-1520. https://doi.org/10.1210/endo-103-4-1518

Rotstein, D.L., Chen, H., Wilton, A.S., Kwong, J.C., Marrie, R.A., Gozdyra, P., Krysko, K.M., Kopp, A., Copes, R., Tu, K. (2018) Temporal trends in multiple sclerosis prevalence and incidence in a large population. *Neurology* 90: e1435-e1441. https://doi.org/10.1212/WNL.0000000000005331

Russel, M. G. V. M., Dorant, E., Volovics, A., Brummer, R.-J., Pop, P., Muris, J. W. M., Bos, L. P., Limonard, C. B. G., Stockbrügger, R. W. (1998) High Incidence of Inflammatory Bowel Disease in The Netherlands. *Diseases of the Colon & Rectum* 41: 33-40. https://doi.org/10.1007/BF02236893

Salhofer-Polanyi, S., Cetin, H., Leutmezer, F., Baumgartner, A., Blechinger, S., Dal-Bianco, A., Altmann, P., Bajer-Kornek, B., Rommer, P., Guger, M., Leitner-Bohn, D., Reichardt, B., Alasti, F., Temsch, W., Stamm, T. (2017) Epidemiology of Multiple Sclerosis in Austria. *Neuroepidemiology* 49: 40-44. https://doi.org/10.1159/000479696

Schober, E., Rami, B., Waldhoer, T., Austrian Diabetes Incidence Study Group (2008) Steep increase of incidence of childhood diabetes since 1999 in Austria. Time trend analysis 1979-

2005. A nationwide study. *European Journal of Pediatrics* 167: 293-297. https://doi.org/10.1007/s00431-007-0480-5

Schonmann, Y., Ashcroft, D. M., Iskandar, I. Y. K., Parisi, R., Sde-Or, S., Comaneshter, D., Batat, E., Shani, M., Vinker, S., Griffiths, C. E. M., Cohen, A. D. (2019) Incidence and prevalence of psoriasis in Israel between 2011 and 2017. *Journal of The European Academy of Dermatology and Venereology* 33: 2075-2081. https://doi.org/10.1111/jdv.15762

Sella, T., Shoshan, A., Goren, I., Shalev, V., Blumenfeld, O., Laron, Z., Chodick, G. (2011) A retrospective study of the incidence of diagnosed Type 1 diabetes among children and adolescents in a large health organization in Israel, 2000-2008. *Diabetic Medicine* 28: 48-53. https://doi.org/10.1111/j.1464-5491.2010.03174.x

Sewerin, P., Brinks, R., Schneider, M., Haase, I., Vordenbäumen (2019) Prevalence and incidence of psoriasis and psoriatic arthritis. *Annals of the Rheumatic Diseases* 78(2): 286-287. https://doi.org/10.1136/annrheumdis-2018-214065

Shapiro, J. M., Zoega, H., Shah, S. A., Bright, R. M., Mallette, M., Moniz, H., Grabert, S. A., Bancroft, B., Merrick, M., Flowers, N. T., Samad, Z., Lidofsky, S., LeLeiko, N. S., Sands, B. E. (2016) Incidence of Crohn's Disease and Ulcerative Colitis in Rhode Island: Report from the Ocean State Crohn's and Colitis Area Registry. *Inflammatory Bowel Disease* 22(6): 1456-1461. https://doi.org/10.1097/MIB.0000000000000745

Sharma, A., Mukewar, S., Czaja, A. (2017) 913. Epidemiology, Clinical Characteristics and Outcomes of Autoimmune Hepatitis in Olmsted County: A Population-Based Study. *The American Journal of Gastroenterology* 112: S511,S513.

Shivashankar, R., Tremaine, W., Harmsen, W.S., Loftus, Jr., E.V. (2017) Incidence and Prevalence of Crohn's Disease and Ulcerative Colitis in Olmsted County, Minnesota From 1970 Through 2010. *Clinical Gastroenterology and Hepatology* 15(6): 857-863. https://doi.org/10.1016/j.cgh.2016.10.039

Sjöberg, D., Holmström, T., Larsson, M., Nielsen, A., Holmquist, L., Ekbom, A., Rönnblom, A. (2013) Incidence and natural history of ulcerative colitis in the Uppsala Region of Sweden 2005-2009 -- Results from the IBD Cohort of the Uppsala Region (ICURE). *Journal of Crohn's and Colitis* 7: e351-e357. https://doi.org/10.1016/j.crohns.2013.02.006

Skov, J., Sundström, A., Ludvigsson, J. F., Kämpe, O., Bensing, S. (2019) Sex-Specific Risk of Cardiovascular Disease in Autoimmune Addison Disease -- A Population-Based Cohort

Study. *The Journal of Clinical Endocrinology & Metabolism* 104: 2031-2040. https://doi.org/10.1210/jc.2018-02298

Spence, R. D., Voskuhl, R. R. (2012) Neuroprotective effects of estrogens and androgens in CNS inflammation and neurodegeneration. *Frontiers in Neuroendocrinology* 33: 105-115. https://doi.org/10.1016/j.yfrne.2011.12.001

Springate, D. A., Parisi, R., Kontopantelis, E., Reeves, D., Griffiths, C. E. M., Ashcroft, D. M. (2017) Incidence, prevalence and mortality of patients with psoriasis: a U.K. population-based cohort study. *British Journal of Dermatology* 176: 650-658. https://doi.org/10.1111/bjd.15021

Stowe, S. P., Redmond, S. R., Stormont, J. M., Shah, A. N., Chessin, L. N., Segal, H. L., Chey, W. Y. (1990) An Epidemiologic Study of Inflammatory Bowel Disease in Rochester, New York. *Gastroenterology* 98: 104-110. https://doi.org/10.1016/0016-5085(90)91297-J

Sundström, P., Nyström, L., Forsgren L. (2003) Incidence (1988-97) and prevalence (1997) of multiple sclerosis in Västerbotten County in northern Sweden. *Journal of Neurology, Neurosurgery, and Psychiatry* 74: 29-32. https://doi.org/10.1136/jnnp.74.1.29

Svensson, J., Lyngaae-Jørgensen, A., Carstensen, B., Simonsen, L.B., Mortensen, H.B. (2009) Long-term trends in the incidence of type 1 diabetes in Denmark: the seasonal variation changes over time. *Pediatric Diabetes* 10: 248-254. https://doi.org/10.1111/j.1399-5448.2008.00483.x

Takahashi, H., Nakamura, K., Kaneko, F., Nakagawa, H., Iizuka, H., Japanese Society for Psoriasis Research (2011) Analysis of psoriasis patients registered with the Japanese Society for Psoriasis Research from 2000-2008. *Journal of Dermatology* 38: 1125-1129. https://doi.org/10.1111/j.1346-8138.2010.01145.x

Taplin, C.E., Craig, M.E.,, Lloyd, M., Taylor, C., Crock, P., Silink, M., Howard, N.J. (2005) The rising incidence of childhood type 1 diabetes in New South Wales, 1990-2002. *Medical Journal of Australia* 183(5): 243-246. https://doi.org/10.5694/j.1326-5377.2005.tb07028.x

Teeäär, T., Liivak, N., Heilman, K., Kool, P., Šor, R., Paal, M., Einberg, Ü., Tillmann, V. (2010) Increasing incidence of childhood-onset type 1 diabetes mellitus among Estonian children in 1999-2006. Time trend analysis 1983-2006. *Pediatric Diabetes* 11: 107-110. https://doi.org/10.1111/j.1399-5448.2009.00535.x

Terao, S., Suzuki, S., Yaita, H., Kurahara, K., Shunto, J., Furuta, T., Maruyama, Y., Ito, M., Kamada, T., Aoki, R., Inoue, K., Manabe, N., Haruma, K. (2020) Multicenter study of autoimmune gastritis in Japan: Clinical and endoscopic characteristics. *Digestive Endoscopy* 32: 364-372. https://doi.org/10.1111/den.13500

Tezel, A., Dökmeci, G., Eskiocak, M., Ümit, H., Soylu, A. R. (2003) Epidemiological Features of Ulcerative Colitis in Trakya, Turkey. *The Journal of International Medical Research* 31: 141-148. https://doi.org/10.1177/147323000303100211

Thunander, M., Petersson, C., Jonzon, K., Fornander, J., Ossiansson, B., Torn, C., Edvardsson, S., Landin-Olsson, M. (2008) Incidence of type 1 and type 2 diabetes in adults and children in Kronoberg, Sweden. *Diabetes Research and Clinical Practice* 82: 247-255. https://doi.org/10.1016/j.diabres.2008.07.022

Tillett, W., Charlton, R., Nightingale, A., Snowball, J., Green, A., Smith, C., Shaddick, G., McHugh, N. (2017) Interval between onset of psoriasis and psoriatic arthritis comparing the UK Clinical Practice Research Datalink with a hospital-based cohort. *Rheumatology* 56: 2109-2113. https://doi.org/10.1093/rheumatology/kex323

Tollefson, M. M., Crowson, C. S., McEvoy, M. T., Kremers, H. M. (2009) Incidence of psoriasis in children: A population-based study. *Journal of the American Academy of Dermatology* 62: 979-987. https://doi.org/10.1016/j.jaad.2009.07.029

Tung, J.Y., Kwan, E.Y., But, B.W., Wong, W.H., Fu, A.C., Pang, G., Tsang, J.W., Yau, H.C., Belaramani, K., Wong, L.M., Wong, S.M., Lo, P., Ng, K.L., Yeung, W.K., Chan, K.T., Chan, A.M., Wong, S.W., Tay, M.K., Chung, J., Lee, C.Y., Lam, Y.Y., Cheung, P.T. (2020) Increasing incidence of type 1 diabetes among Hong Kong children and adolescents: The Hong Kong Childhood Diabetes Registry 2008 to 2017. *Pediatric Diabetes* 21(5):713-719. https://doi.org/10.1111/pedi.13016

Turunen, P., Kolho, K.-L., Auvinen, A., Iltanen, S., Huhtala, H., Ashorn, M. (2006) Incidence of Inflammatory Bowel Disease in Finnish Children, 1987-2003. *Inflammatory Bowel Diseases* 12(8): 677-683. https://doi.org/10.1097/00054725-200608000-00002

van Gerven, N. M. F., Verwer, B. J., Witte, B. I., van Erpecum, K. J., van Buuren, H. R., Maijers, I., Visscher, A. P., Verschuren, E. C., van Hoek, B., Coenraad, M. J., Beuers, U. H. W., de Man, R. A., Drenth, J. P. H., den Ouden, J. W., Verdonk, R. C., Koek, G. H., Brouwer, J. T., Guichelaar, M. M. J., Vrolijk, J. M., Mulder, C. J. J., van Nieuwkerk, C. M.

J., Bouma, G. (2014) Epidemiology and clinical characteristics of autoimmune hepatitis in the Netherlands. *Scandinavian Journal of Gastroenterology* 49(10): 1245-1254. https://doi.org/10.3109/00365521.2014.946083

van Paassen, P., Vriesman, P., van Rie, H., Tervaert, J. (2004) Signs and symptoms of this basement membrane nephropathy: A prospective regional study on primary glomerular disease--The Limburg Renal Registry. *Kidney International* 66: 903-913. https://doi.org/10.1111/j.1523-1755.2004.00835.x

Vehik, K., Hamman, R.F., Lezotte, D., Norris, J.M., Klingensmith, G., Bloch, C., Rewers, m., Dabelea, D. (2007) Increasing Incidence of Type 1 Diabetes in 0- to 17-Year-Old Colorado Youth. *Diabetes Care* 30(3): 503-509. https://doi.org/10.2337/dc06-1837

Warren, S., Svenson, L., Warren, K., Metz, L., Patten, S., Schopflocher, D. (2007) Incidence of Multiple Sclerosis among First Nations People in Alberta, Canada. *Neuroepidemiology* 28: 21-27. https://doi.org/10.1159/000097852

Weets, I., De Leeuw, I.H., Du Caju, M.V.L., Rooman, R., Keymeulen, B., Mathieu, C., Rottiers, R., Daubresse, J.-C., Rocour-Brumioul, D., Pipeleers, D.G., Gorus, F.K. (2002) The Incidence of Type 1 Diabetes in the Age Group 0-39 Years Has Not Increased in Antwerp (Belgium) Between 1989 and 2000. *Diabetes Care* 25(5): 840-846. https://doi.org/10.2337/diacare.25.5.840

Weng, J., Zhou, Z., Guo, L., Zhu, D., Ji, L., Luo, X., Mu, Y., Jia, W. (2018) Incidence of type 1 diabetes in China, 2010-13: population-based study. *British Medical Journal* 360: j5295. https://doi.org/10.1136/bmj.j5295

Werner, M., Prytz, H., Ohlsson, B., Almer, S., Björnsson, E., Bergquist, A., Wallerstedt, S., Sandberg-Gertzén, H., Hultcrantz, R., Sangfelt, P., Weiland, O., Danielsson, Å. (2008) Epidemiology and the initial presentation of autoimmune hepatitis in Sweden: A nationwide study. *Scandinavian Journal of Gastroenterology* 43(10): 1232-1240. https://doi.org/10.1080/00365520802130183

West, J., Fleming, K., Tata, L., Card, T., Crooks, C. (2014) Incidence and Prevalence of Celiac Disease and Dermatitis Herpetiformis in the UK Over Two Decades: Population-Based Study. *The American Journal of Gastroenterology* 109: 757-768. https://doi.org/10.1038/ajg.2014.55

Westcott, P. M., Halliwill, K. D., To, M. D., et al. (2015) The mutational landscapes of genetic and chemical models of Kras-driven lung cancer. *Nature* 517: 489-492. https://doi.org/10.1038/nature13898

Willis, J.A., Scott, R.S., Darlow, B.A., Nesbit, J.W., Anderson, P., Moore, M.P., Lunt, H., Cole, D.R. (2002) Incidence of Type 1 Diabetes Mellitus Diagnosed Before Age 20 Years in Canterbury, New Zealand over the last 30 Years. *Journal of Pediatric Endocrinology & Metabolism* 15: 637-643. https://doi.org/10.1515/jpem.2002.15.5.637

Winsa, B., Adami, H.-O., Bergström, R., Gamstedt, A., Dahlberg, P. A., Adamson, U., Jansson, R., Karlsson, A. (1991) Stressful life events and Graves' disease. *The Lancet* 338: 1475-79. https://doi.org/10.1016/0140-6736(91)92298-g

Wynn, D. R., Rodriguez, M., O'Fallon, W. M., Kurland, L. T. (1990) A reappraisal of the epidemiology of multiple sclerosis in Olmsted County, Minnesota. *Neurology* 40:780-786. https://doi.org/10.1212/WNL.40.5.780

Yang, S-K., Hong, W-S., Min, Y., Kim, H., Yoo, J., Rhee, P-L., Rhee, J., Chang, D., Song, I., Jung, S., Park, E-B., Yoo, H., Lee, D., Kim, Y. (2001) Incidence and prevalence of ulcerative colitis in the Songpa-Kangdong District, Seoul, Korea, 1986-1997. *Journal of Gastroenterology and Hepatology* 15: 1037-1042. https://doi.org/10.1046/j.1440-1746.2000.02252.x

Yoshizawa, K., Joshita, S., Matsumoto, A., Umemura, T., Tanaka, E., Morita, S., Maejima, T., Ota, M. (2016) Incidence and prevalence of autoimmune hepatitis in the Ueda area, Japan. *Hepatology Research* 46: 878-883. https://doi.org/10.1111/hepr.12639

Zeller, B., Helgestad, J., Hellebostad, M., Kolmannskog, S., Nystad, T., Stensvold, K. (2000) Immune Thrombocytopenic Purpura in Childhood in Norway: A Prospective, Population-Based Registration. *Pediatric Hematology and Oncology* 17(7): 551-558. https://doi.org/10.1080/08880010050122816

Zhai, Y., Haresi, A. J., Huang, L., Lang, D. (2020) Differences in tumor initiation and progression of melanoma in the Braf(CA);Tyr-CreERT2;Pten(f/f) model between male and female mice. *Pigment Cell Melanoma Research* 33: 119-121. https://doi.org/10.1111/pcmr.12821

Zhang, H., Jin, Z., Cui, R., Ding, S., Huang, y., Zhou, L. (2017) Autoimmune metaplastic atrophic gastritis in Chinese: a study of 320 patients at a large tertiary medical center.

Scandinavian Journal of Gastroenterology 52(2): 150-156. https://doi.org/10.1080/00365521.2016.1236397

Zhao, F., Wu, L., Wang, Y., et al. (2019) Dihydrotestosterone regulates oxidative stress and immunosuppressive cytokines in a female BALB/c mouse model of Graves' disease. *Autoimmunity* 52: 117-125. https://doi.org/10.1080/08916934.2019.1621857

Zhao, Z., Sun, C., Wang, C., Li, P., Wang, W., Ye, J., Gu, X., Wang, X., Shen, S., Zhi, D., Lu, Z., Ye, R., Cheng, R., Xi, L., Li, X., Zheng, Z., Zhang, M., Luo, F. (2014) Rapidly rising incidence of childhood type 1 diabetes in Chinese population: epidemiology in Shanghai during 1997-2011. *Acta Diabetologica* 51: 947-953. https://doi.org/10.1007/s00592-014-0590-2
